# The application of network agenda setting model during the COVID-19 pandemic based on latent dirichlet allocation topic modeling

**DOI:** 10.3389/fpsyg.2022.954576

**Published:** 2022-09-27

**Authors:** Kai Liu, Xiaoyu Geng, Xiaoyan Liu

**Affiliations:** ^1^School of Languages and Communication Studies, Beijing Jiaotong University, Beijing, China; ^2^Guangming School of Journalism and Communication, China University of Political Science and Law, Beijing, China

**Keywords:** agenda setting, LDA topic modeling, agenda network, implicit public agenda, explicit public agenda

## Abstract

Based on Network Agenda Setting Model, this study collected 42,516 media reports from Party Media, commercial media, and We Media of China during the COVID-19 pandemic. We trained LDA models for topic clustering through unsupervised machine learning. Questionnaires (*N* = 470) and social network analysis methods were then applied to examine the correlation between media network agendas and public network agendas in terms of explicit and implicit topics. The study found that the media reports could be classified into 14 topics by the LDA topic modeling, and the three types of media presented homogeneity in the topics of their reports, yet had their own characteristics; there was a significant correlation between the media network agenda and the public network agenda, and the We Media reports had the most prominent effect on the public network agenda; the correlation between the media agenda and the implicit public agenda was higher than that of the explicit public agenda. Overall, findings showed a significant correlation between network agendas among different media.

## Introduction

The COVID-19 pandemic has had a significant impact on all aspects of society from the start, and almost every member of the public has been affected. Many participated in discussions on the Internet on topics related to the pandemic in numerous ways, expressing their emotions and opinions. Whether the media and the public share the same focus on a topic—in this case, the pandemic—and whether there has been a cross influence between them is a core focus of agenda-setting theory. Studies have shown that there is a high correlation between the key topics that media and the public choose to focus on.

Attribute differences of issues were later introduced into network-agenda setting theory, such as the commonly used issue classifications of “substantive attribute agenda” and “affective attribute agenda.” McCombs et al. (1997) analyzed data from a study of the 1995 Spanish regional municipal elections and found that the attribute agenda setting and the affective attribute agenda of candidates presented in the mass media influenced the attribute agenda of candidates’ image among voters. Subsequently, Golan and Wanta (2001) also found in their survey data from the 2000 U.S. presidential election in New Hampshire that the attribute agenda mentioned in newspaper reports influenced voters’ judgments about the attributes of the two Republican candidates.

The above two types of theoretical perspectives begin with two types of variables—media types and agenda attributes—to examine the interaction between public and media perceptions of an issue. However, not all media have the same influence, and the Internet environment is more complex. Traditional media leadership has been particularly challenged by the rise of social media. Communication scholars are beginning to pay more attention to the public’s ability to upend the media agenda. Wang et al. (2021) found that agenda-setting for wildlife-related issues on Weibo did not follow a one-way path from media to audience, but instead exhibited a reciprocal, dynamic interaction during the COVID-19 pandemic.

Network agenda-setting during the COVID-19 pandemic has also been challenging. First, the pandemic has involved and affected almost every member of the public, and the influence and scope of public agenda setting are more extensive. Second, types of media are also more complex in the Internet environment, for example, We Media, influencers, and Internet celebrities have played important roles in agenda-setting regarding the pandemic. Finally, the pandemic prompted the media to push out information quicker and break the “cocoon” between communities, enabling individuals to pay attention to other groups. In a sense, it has facilitated community-to-community connections. Based on the above three realities, this paper asks the research question: what kind of network structure characteristics do the agendas among media, between media and public, and among different types of issues present in the coverage of the COVID-19 pandemic?

This study focuses on and explores several aspects. First, theoretically, the cross-interaction relationship between media and public agendas is examined from Network Agenda Setting Model (NAS) in the case of the COVID-19 pandemic. Second, in terms of methodology, machine learning methods were used to process the massive amount of public and media agenda-setting data. Recent literature has recognized that computer-assisted textual analysis can allow for a more holistic picture to be obtained when working with a large volume of text data (Gong and Firdaus, 2022). Compared to traditional content analysis, this approach increases the efficiency of text classification tasks. Furthermore, in terms of media type, existing research has focused on the role played by mainstream media yet neglected the power of We Media. Some studies have shown that official media failed to lead the discussion, whereas commercial media tended to be more influential than in the past in crisis cases (Wang and Shi, 2022). Whether this conclusion is also able to be proven in the pandemic is still a question. Therefore, in the current study, we further broke down media into three specific types: Party Media, commercial media, and We Media.

## Theoretical background

### Network agenda setting model

Network agenda setting (NAS) theory was first proposed by Guo and McCombs (2011) to explore the interaction relationship between media and public agenda networks. Over time the theory has developed to become widely applied in the analysis of the issue network relationship between traditional media and social media. Vargo et al. (2014) analyzed a large Twitter dataset using a series of methods, verifying that different audiences “melded” the agendas of various media in distinctly different ways. Kweon et al. (2019) found that media agenda-setting functions have an increasing influence on public perceptions of social network sites in the context of economic issues in Korea.

More recently, further variables have been taken into account in the exploration of NAS. For instance, Wang (2016) integrated crisis and non-crisis news into the network. Meanwhile, country differences have also been introduced as important variables in related studies. Guo et al. (2019) examined news coverage of the South China Sea dispute on Twitter in three countries, China, the U.S., and the Philippines. Su and Hu (2020) examined the issue of the Diaoyu Islands dispute as reported in Chinese, Japanese, and American newspapers as well as on Twitter and studied the mediating effects between newspapers and Twitter. Scholars have also examined the reliability, validity, and effectiveness of NAS in non-Western contexts.

The theory of NAS has been gradually introduced into other fields beyond political science, and the theoretical horizon is expanding accordingly. Guo and Vargo (2015) integrated NAS with issue ownership theory and proposed the concept of an issue ownership network. In the field of science communication, Chen and Zhang (2021) adopted the NAS model to delineate the salient attributes of gene-editing and their networks in the online agendas of gene-editing and to investigate the interactions between different actors’ agendas.

In addition to applying NAS theory in different research fields, scholars have also used it to explore network characteristics. However, few NAS studies have combined substantive and affective attributes to explore their interactions with the public agenda.

### Media network agenda interaction in different stages of the event

Inter-media agenda-setting is concerned with the interaction of agendas between different types of media, focusing on their mutual influencing roles (Vargo and Guo, 2017). For a long time, research has focused on the differences between traditional media, which is dominated by elite journalists, and social media, which is dominated by the public. McCombs (2005) argued that elite journalists have special power in the process of inter-media agenda setting. Vu et al. (2014) tested 5 years (2007–2011) of aggregate data from national news media and polls in the U.S., and found a high degree of similarity in issue networks across media, including newspapers, radio, television, and online news media. Vargo and Guo (2017) studied online media sources in the U.S. in 2015, and their modeling suggests that media agendas are highly homogeneous and complementary, with elite newspapers no longer in control of the news agenda, the public preferring to follow online partisan media, and online partisan media replacing elite newspapers in holding a dominant role in the overall media agenda.

In addition to examining traditional and social media differences, Weaver et al. (2010) identified another two ways to consider media, vertical and horizontal, which have been gradually incorporated into current research. Vertical media refers to media that radiates to all segments of society, while horizontal media focus on people with specific interests and expertise. Guo and Vargo (2015) examined the 2012 U.S. presidential election using an online agenda-setting model, and confirmed that Obama supporters tended to follow the network agenda of vertical mainstream media, whereas Romney supporters were more in line with the conservative niche media—horizontal media.

These two media segmentation methods, however, do not consider the impact of the different stages of an event. Sung and Hwang (2014) found that in the field of crisis communication, although social media may play an important role as a major news source during the initial stage, once traditional news reports appear, these take the lead in setting the agenda. Significant differences were also observed in the influence of agenda-setting by various media at different stages of the COVID-19 pandemic in China. In the early stage of the outbreak, mainstream media, with their resource advantages, were able to take the lead in first-line reporting, providing the public with timely and accurate information. This shows that vertical media had a greater influence on the agenda over horizontal media at the start. Meanwhile, in the subsequent stages of the pandemic, the focus of We Media seemed to profoundly influence the issues of traditional media. Generally, We Media is better able to report on social issues affecting niche or vulnerable groups. These issues have also been of great concern to the public as the pandemic has reached it later stages. Based on this preliminary observation, the current study takes the event progression process as an important variable reference to examine whether there is a correlation between the agenda networks among media at different stages. Therefore, this paper proposed the following hypothesis.

*Hypothesis 1 (H1)*: Regarding the media coverage of the COVID-19 pandemic, there was a significant correlation between the network agendas of different types of media in the different stages of the pandemic development.

### Interactive relationship between media network agenda and public agenda: From linear influence to the network influence

Media influence on public issues occupies a large proportion of existing studies on the relationship between the media network agenda and the public agenda. Schultz et al. (2012) found that institutions can reverse public attention and issue-focused perceptions through the media, while Guo and Vargo (2015) showed that new media can influence public perceptions not only with individual issues but also with the network relationship between issues. But this relationship is not unidirectional. Within the context of the Internet environment, the influence between public agendas and media agendas is mutual. Weiss-Blatt (2016) found a positive correlation between the salience of technology-based coverage issues in traditional media coverage and blogs written by the public. However, due to media network agendas, audiences do not always hear about all current issues as the process of agenda-setting is influenced by many individual factors, such as individual demographic differences, media exposure, user needs, and partisan stances.

Continuing on from the above theory and findings, the issue of the COVID-19 pandemic is particularly complex with numerous considerations one must consider, and its social impact has had a particularly large scope. The agenda relationship between the public and the media has more “network” characteristics than linear influences. The agendas surrounding the COVID-19 pandemic have been relevant to everyone, and the public’s perception of this issue is an important variable to be examined. Therefore, in this study, issue differences are placed as important variables in the relationship between the two.

### Network agenda under the differences between explicit agendas and implicit agendas

The NAS model posits the existence of two levels of public agendas: implicit and explicit (Guo, 2012). “Implicit” here means unconscious, indirect, and automatic. Individuals may not even realize when they unconsciously connect two things in their minds. In contrast, an explicit public agenda refers to a conscious, active connection. Some studies have found that media agendas are more closely associated with the public’s implicit agendas. This view was verified in the study of Jiang and Cheng (2018), who found that newspaper agendas were found to be more closely associated with implicit public agendas. Subsequently, Jiang et al. (2021) validated the NAS model in multiple social contexts. In analyzing the THAAD event (the deployment of the Terminal High Altitude Area Defense system in South Korea), research data from China, the United States, and South Korea showed that the NAS effect on the implicit public agenda was stronger than its corresponding effect on the explicit public agenda. A possible explanation for this could be that when the media agenda transmitted messages to the public, individuals might not have explicitly realized the connection between the any two attributes they have made and were thus more quickly influenced by the messages coming *via* the implicit media agenda.

By breaking down the explicit and implicit associations in the public agenda, it is possible to more closely examine how and at what level the media agenda influences public thought (Vargo et al., 2014). Therefore, this study proposes the following hypotheses based on the above findings.

*Hypothesis 2 (H2)*: There was a significant correlation between the media network agenda and the explicit public agenda regarding media coverage of the COVID-19 pandemic.

*Hypothesis 3 (H3)*: There was a significant correlation between the media network agenda and the implicit public agenda regarding media coverage of the COVID-19 pandemic.

## Research methods

### Subjects and data

#### Media agendas

In this study, three types of media were selected as research subjects according to the characteristics of China media: Party Media,^1^ commercial media (Southern Metropolis Daily), and We Media.^2^ As Party media, ChinaNews.com is one of the top 10 most important news organizations in China; as mainstream media with a high degree of marketization, Southern Metropolis Daily has influence nationally; and finally, the reports collected from the official cn-healthcare.com website were from “health” channel, which is a popular health We Media platform. The platform invites a large number of content creators—most of which are medical practitioners—to share health policies, disease knowledge, and other health-related content. All three media ranked first in their number of reports about the outbreak of the pandemic in their particular media category, ensuring that they were influential media players. The data collected in relation to the COVID-19 pandemic were obtained from the WiseNews database, with data volumes of 23,452 for ChinaNews.com, 11,902 for Southern Metropolis Daily, and 7,162 for cn-healthcare.com.

#### Public agendas

Questionnaires were the primary way used to collect data regarding public agendas. First, focus groups were used as pre-studies to supplement the questionnaire design. Focus group interviews were conducted to obtain public perceptions of the COVID-19 pandemic. Based on the narratives in the focus group interviews, we compiled a list of topics that made up the public agenda. Comparing the topics from the focus group interviews with the media report topics, a large number of synonymous or near-synonymous expressions were found, so a final list of 14 topics were integrated into the content analysis that was used in designing the questionnaire. Considering participants’ gender and age differences, the focus groups were divided into in four groups, with six participants in each group (see Table 1).

**Table 1:** Focus group.

Focus group	Number of participants	Gender	Age
1	6	3Males + 3females	18–23
2	6	3Males + 3females	24–29
3	6	3Males + 3 females	30–35
4	6	3Males + 3 females	36–40

The study used the mind-mapping method to collect data for both the implicit and explicit network public agendas in the questionnaire. In the first part, respondents selected certain topics from the full list of topics to test for implicit associations. In the second part, respondents selected related topics from the selected topics and established a link between two to test the explicit associations.

The questionnaire was distributed through the wjx.cn platform from April 26, 2021 to May 11, 2021. The public was still in the midst of the pandemic during this period, so we were able to observe the impact of the media agenda on the public. To proportionally represent Chinese netizens, the current study refers to survey data from the 47th Statistical Report on Internet Development in China released by the CNNIC in February 2021 for sampling based on gender, age, and education (China Internet Network Information Center, 2021). About 470 participants completed the survey, and 240 participants (51%) were male, and 230 (49%) were female. The mean age of participants was 29.3.In terms of education level, 40.4% had high school education or below, while 59.56% held a bachelor’s degree or higher.

### Definition of the main variables

#### The basis for the stage division of the pandemic

**Figure 1:**
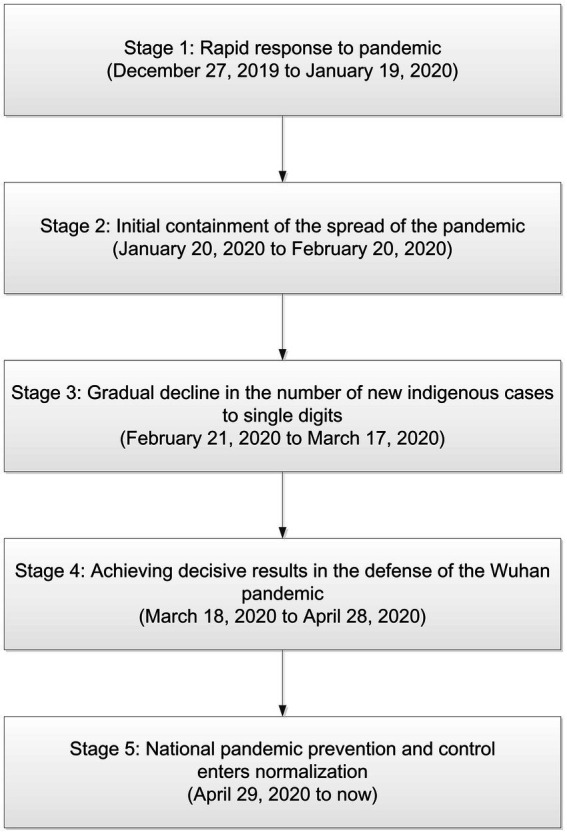
Five stages of fighting COVID-19 in China.

According to the “Fighting COVID-19 China in Action” White Paper issued by the State Council Information Office of the People’s Republic of China, China’s arduous journey through the pandemic can be divided into five stages, as shown in Figure 1.The study focused on the time interval from December 27, 2019 to April 28, 2020, with consideration that the pandemic was in its normalization phase from April 29, 2020 to April 29, 2021.

#### Classification of explicit and implicit issues

The explicit and implicit measurements in NAS have been a topic of discussion in academic circles. Currently explicit and implicit measures can be divided into “mind mapping” and “elaboration.” In this study, the “mind mapping” method was used to measure explicit and implicit associations. In practice, this means that if participants select words from a word list, they are considered to have an implicit relationship with each other, while if participants construct connections between these words by drawing lines, the words marked by the lines are considered to be explicitly related (Jiang and Cheng, 2018). In our study, as part of the questionnaire design, participants were first asked to select up to 10 words from the topic list to describe COVID-19 pandemic, and co-occurrences of the selected words were used to measure the implicit association between the attributes; for the explicit association, participants connected the attribute words they considered to be related to each other by drawing lines, and the lines clearly marked the explicit association between the attributes.

### Main analysis tools

#### Machine learning and LDA topic modeling

**Figure 2:**
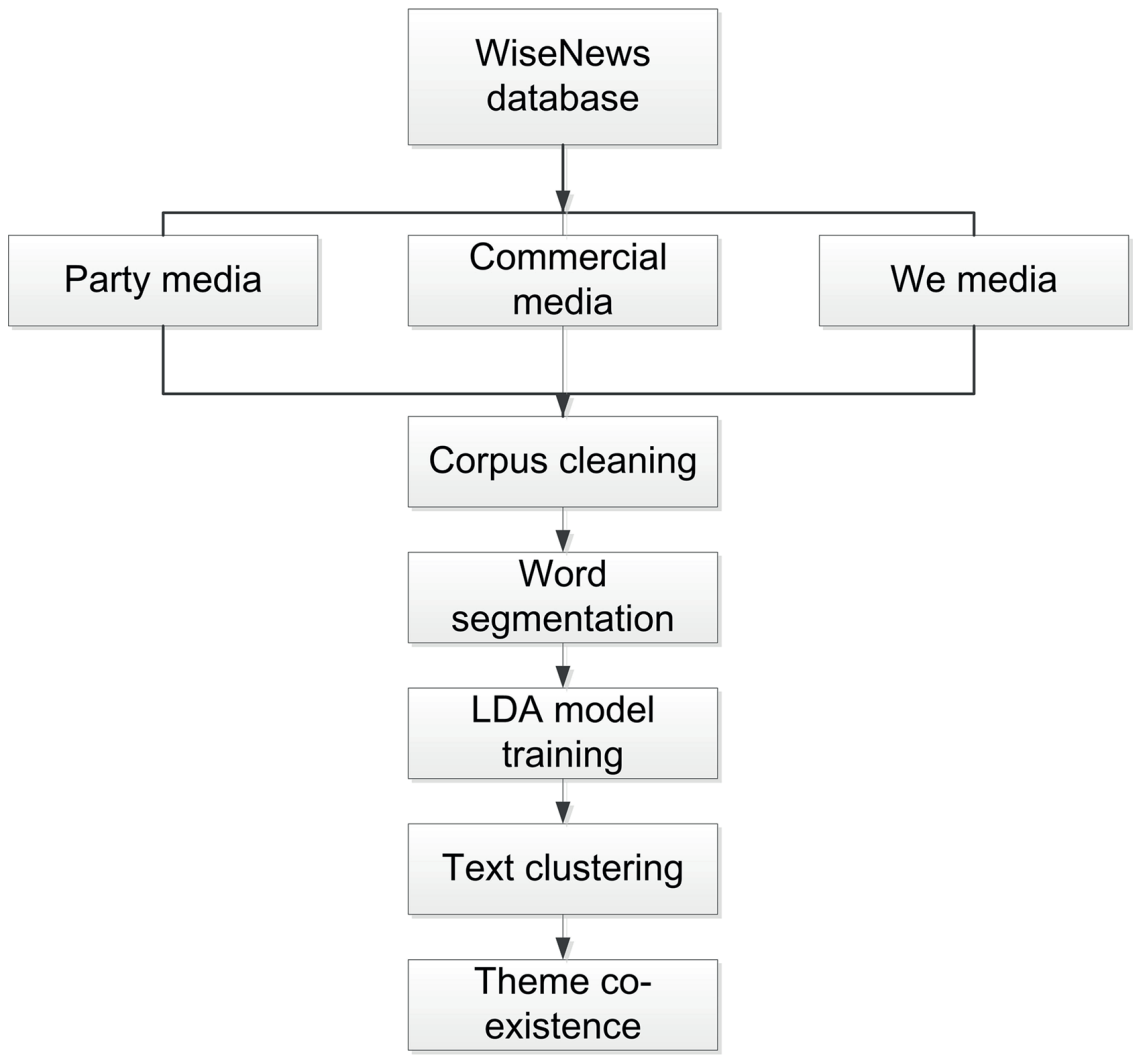
Analysis process.

In this paper, we used unsupervised machine learning—Latent Dirichlet Allocation (LDA) Topic Modeling—for big data analysis using Python. The LDA model can automatically generate text collections to identify salient topics in news stories, and existing research has proven that LDA database-based topic modeling can effectively discover and understand the underlying topic structure for topic analysis (Guo et al., 2016). The analysis process is shown in Figure 2, where the pre-processing of different news corpus was performed using the Chinese word splitting tool “jieba,” setting custom dictionaries to add words specific to the pandemic context (e.g., names of people, places, drugs, etc.), set deactivation words, and filter out useless words. For the LDA Topic Modeling, all news articles in the three media types were trained using the Python three-way library “genism.” With reference to fixed metrics such as perplexity, topic coherence, and multiple training sessions, the *k*-value interval was calculated approximately. The optimal result was determined by trying each of the *k*-values in this interval, resulting in 14 generated topics. LDA Topic Modeling can calculate the topic probability distribution of each article based on the trained topics, which provides the conditions for co-occurrence between topics. By calculating the *k*-value set to 14, the average value of each topic probability was 0.07. At this point the probability of two or more topics in a report was greater than or equal to 0.14, and these topics would then be considered to be one co-occurrence. The topics in each report that met this requirement were retained. The final step was to build the topic co-occurrence matrix by the matrix function.

#### QAP analysis

Quadratic Assignment Procedure (QAP) calculates the measures of nominal, ordinal, and interval association between the relations in two matrices, and uses quadratic assignment procedures to develop standard errors to test for the significance of associations (Hanneman and Riddle, 2005). In this paper, QAP analysis was conducted using UCINET to test whether there was a network correlation between media network agendas and public agendas. Finally, the study used NetDraw in UCINET to visualize and analyze the agenda network. The iterative metric multidimensional scaling method was used to map the agenda network, in which the closer that attributes are to one another, the stronger the correlation between the two, and the closer an attribute is connected to other attributes, the closer that attribute is to the location of the center of the network (Jiang and Cheng, 2018).

## Results

### Inter-media network agenda-setting effect

As no reports were retrieved for the first stage of the pandemic (December 27, 2019 to January 19, 2020), only data for the second stage (January 20, 2020 to February 20, 2020), the third stage (February 21, 2020 to March 17, 2020), and the fourth stage (March 18, 2020 to April 28, 2020) are covered.

For H1, with regard to media coverage of the COVID-19 pandemic, there was always a significant correlation between network agendas of different media types as the pandemic developed into different stages. The results demonstrated statistically significant associations between network agendas of different media (see Table 2). Specifically, QAP correlation results showed that at all three stages, the agendas of any of the two media were significantly correlated, with the highest correlation coefficients for Southern Metropolis Daily and ChinaNews.com (Pearson’s *r* = 0.924, 0.929, and 0.94, respectively, *p* < 0.001 for all three stages). Cn-healthcare.com and ChinaNews.com had the lowest correlation coefficients in the three stages (Pearson’s *r* = 0.655, 0.691, and 0.668, respectively, *p* < 0.001 for all three stages). H1 was supported. This suggests that in the new media era, there is a correlational influence of agendas among different media with some homogeneity.

**Table 2:** Effectiveness of agenda setting different media.

Correlation coefficient (*r*)	Stage II	Stage III	Stage IV
Cn-healthcare.com & Southern Metropolis Daily	0.789^***^	0.823^***^	0.775^***^
Cn-healthcare.com & ChinaNews.com	0.655^**^	0.691^**^	0.668^**^
Southern Metropolis Daily & ChinaNews.com	0.924^***^	0.929^***^	0.94^***^

** *p* < 0.01 and

*** *p* < 0.001.

### Interaction between media network agendas and public network agendas

With regard to H2, there was a significant correlation between the media network agenda and the explicit public agenda. QAP correlation results showed statistically significant associations between media network agendas and explicit public network agendas (Pearson’s *r* = 0.381, *p* < 0.01; see Table 3). H2 was supported.

**Table 3:** Effectiveness of network agenda-setting during the COVID-19 pandemic.

Correlation coefficient (*r*)	Explicit Public Agenda	Implicit Public Agenda
Media Network Agenda	0.381^**^	0.521^*^

* *p* < 0.05 and

** *p* < 0.01.

For H3, there was a significant correlation between the media network agendas and the implicit public agendas regarding media coverage of the COVID-19 outbreak. QAP correlation results showed that statistically significant associations between media network agendas and implicit public network agendas (Pearson’s *r* = 0.521, *p* < 0.05; see Table 3). H3 was supported.

Quadratic Assignment Procedure correlation results showed that the coefficient of cn-healthcare.com was the highest (Pearson’s *r* = 0.364, *p* < 0.01), and the coefficient of ChinaNews.com was the lowest (Pearson’s *r* = 0.308, *p* < 0.01) between media agenda and explicit public agenda (see Table 4).

**Table 4:** Effectiveness of agenda-setting in different media networks.

Correlation coefficient (*r*)	Explicit Public Agenda	Implicit Public Agenda
Cn-healthcare.com	0.364^**^	0.658^**^
Southern Metropolis Daily	0.325^**^	0.361
ChinaNews.com	0.308^**^	0.305

** *p* < 0.01.

Only cn-healthcare.com was significantly associated with the implicit public agenda (Pearson’s *r* = 0.658, *p* < 0.01), confirming the professional role played by medical We Media in the COVID-19 pandemic.

### Media agenda network during the COVID-19 pandemic

#### Homogeneity and individuality of media network agenda

The LDA algorithm was used to extract 14 topics from the total sample (42,516 reports) to build a co-occurrence matrix. Topic of “Supply of materials such as masks” accounted for the largest proportion at 16.31%, followed by the topic of “Healthcare workers” (12.73%) and “government policy”(11.79%). And the degree centrality was calculated using UCINET for ranking and visualization. The media reports were related to the supply of materials, such as masks (426,273), healthcare workers (422,823), government policy (400,098), community prevention and control (396,651), virus analysis (378,335), social mobilization (367,750), overseas outbreaks (364,082), vaccine development (356,496), support Wuhan (353,602), anti-pandemic measures (346,614), global cooperation (305,707), case reports (218,521), overseas input (197,075), and Chinese medical treatment (158,693; See Table 5).

**Table 5:** The 14 topics related to the COVID-19 pandemic.

**Serial number**	**Topics**	**Keywords**	**Proportion**	**Degree centrality**
1	Supply of materials such as masks	Mask, production, enterprise, Supplies, epidemic, medical, need, price, supply, and purchase	16.31%	426,273
2	Healthcare workers	Wuhan, first line, medical staff, hope, anti-epidemic, war, epidemic, protective suit, hold fast, and hero	12.73%	422,823
3	Government policy	Epidemic, prevention and control, assurance, policy, measure, resumption of work and production, implement, introduced, publish, and employment	11.79%	400,098
4	Community prevention and control	Community, prevention and control, quarantine, detect, troubleshoot, manage, resident, street, staff member, and grassroots	11.35%	396,651
5	Virus analysis	Infect, virus, pneumonia, spread, wild animals, infectious disease, expert, game, symptom, and respiratory tract	8.59%	378,335
6	Social mobilization	Donate, win, blockade, xi jinping, party member, the masses, strength, love, fight, and war epidemic	7.77%	367,750
7	Overseas outbreaks	America, Japan, Confirmed, Germany, Italy, France, government, Overseas Chinese, South Korea, and International students	6.95%	364,082
8	Vaccine development	Vaccine, detect, research, R&D, clinical, technology, biology, scientific research, antibody, and laboratory	6.15%	356,496
9	Support Wuhan	Hospital, patient, Wuhan, treat, medical team, support, medical staff, mobile cabin hospitals, players, and expert group	5.22%	353,602
10	Anti-pandemic measures	Mask, disinfect, body temperature, protection, wear, touch, ventilation, register, gather, and home	5.06%	346,614
11	Global cooperation	China, economy, worldwide, nation, cooperate, develop, crisis, cope with, market, and public health	3.33%	305,707
12	Case reports	Case, confirmed, add, grand total, die, decline, data, cure, suspected case, and continuous	3.14%	218,521
13	Offshore input	Traveler, airport, flight, customs, port, entry, input, quarantine, fever, and medical observation	1.58%	197,075
14	Chinese medicine treatment	Traditional Chinese Medicine, medicinal glue, oral liquid, and shuanghuanglian	0.05%	158,693

In general, all three media types presented homogeneity in reported themes, with all prioritizing “supply of masks and other materials” and “healthcare workers.” As shown in Table 6, the third-ranked topic in the cn-healthcare.com was “virus analysis,” which focused on professional medical issues in the public health vertical, while the Southern Metropolis Daily focused on “community prevention and control,” and on residents’ lives and grassroots management, reflecting the professionalism of the media. Meanwhile, ChinaNews.com mainly reported on government policies and measures, and actively relayed government programs, reflecting the media’s attribute as the mouthpiece of the Party. While other media paid little attention to “overseas outbreaks,” while ChinaNews.com paid more attention to the status of the pandemic in various countries around the world and the lives of Chinese people living overseas.

**Table 6:** Topic ranking in different media.

Ranking	Overall	Cn-healthcare.com	Southern Metropolis Daily	ChinaNews.com
1	Supply of materials such as masks	Supply of materials such as masks	Supply of materials such as masks	Supply of materials such as masks
2	Healthcare workers	Healthcare workers	Healthcare workers	Healthcare workers
3	Government policy	Virus analysis	Community prevention and control	Government policy
4	Community prevention and control	Support Wuhan	Government policy	Community prevention and control
5	Virus analysis	Government policy	Vaccine development	Overseas outbreaks
6	Social mobilization	Vaccine development	Virus analysis	Virus analysis
7	Overseas outbreaks	Community prevention and control	Social mobilization	Social mobilization
8	Vaccine development	Anti-pandemic measures	Overseas outbreaks	Anti-pandemic measures
9	Support Wuhan	Social mobilization	Support Wuhan	Support Wuhan
10	Anti-pandemic measures	Global cooperation	Anti-pandemic measures	Vaccine development
11	Global cooperation	Overseas outbreaks	Global cooperation	Global cooperation
12	Case reports	Case reports	Chinese medicine treatment	Offshore input
13	Chinese medicine treatment	Offshore input	Offshore input	Chinese medicine treatment
14	Offshore input	Chinese medicine treatment	Case reports	Case reports

#### Network structure of tripartite confrontation

**Figure 3:**
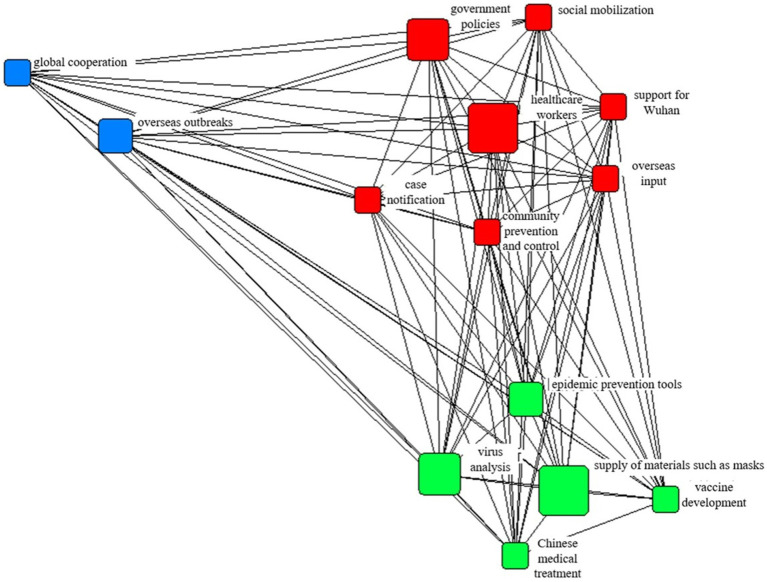
The agenda network of media coverage.

Figure 3 shows the agenda network of media coverage, and the 14 topics were further grouped into three major categories according to the relevance of the content. The first category was prevention and treatment, including “virus analysis,” “vaccine development,” “anti-pandemic measures,” “Chinese medical treatment,” and “supply of masks and other materials.” The second category was the status of the pandemic domestically, including “case reports,” “offshore input,” “healthcare workers,” “support Wuhan,” “government policy,” “community prevention and control,” and “social mobilization.” The third category was the global fight against the pandemic, and included “global cooperation” and “overseas outbreaks.” These three categories of topics show a three-legged feature in terms of network characteristics. Cn-healthcare.com paid more attention to the content in the first category, while Southern Metropolis Daily and ChinaNews.com paid more attention to the content in the second category.

### Features of the public network agenda during the COVID-19 pandemic

**Figure 4:**
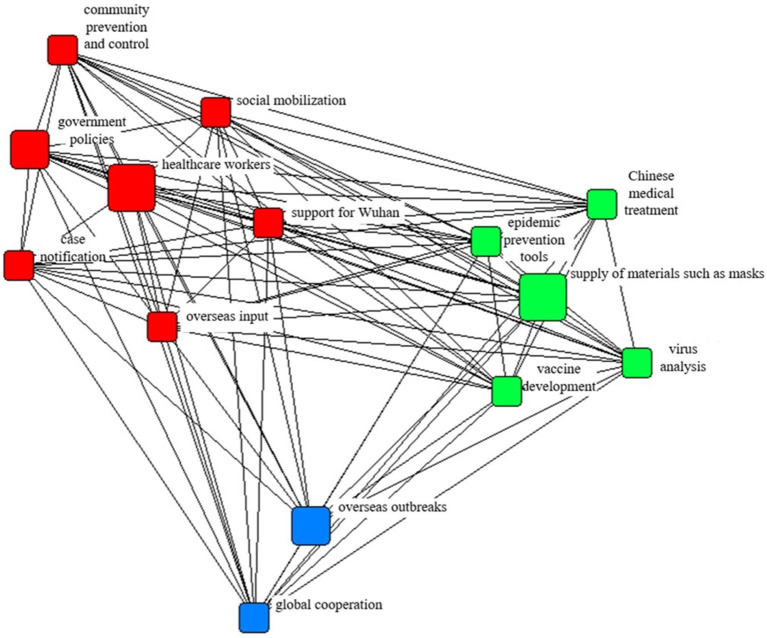
The network of the explicit public agenda.

Figure 4 shows the network visualization of the explicit public agenda during the COVID-19 pandemic. According to the degree centrality as noted in Table 7, the central theme was “supply of masks and other materials” (2,357), followed by “community prevention and control” (844), and “anti-pandemic measures” (783).

**Table 7:** Explicit public agenda topic ranking.

Ranking	Topics	Degree centrality
1	Supply of materials such as masks	2,357
2	Community prevention and control	844
3	Anti-pandemic measures	783
4	Global cooperation	734
5	Virus analysis	688
6	Healthcare workers	650
7	Overseas outbreaks	636
8	Offshore input	492
9	Government policy	478
10	Chinese medicine treatment	443
11	Vaccine development	433
12	Case reports	410
13	Support Wuhan	314
14	Social mobilization	214

**Figure 5:**
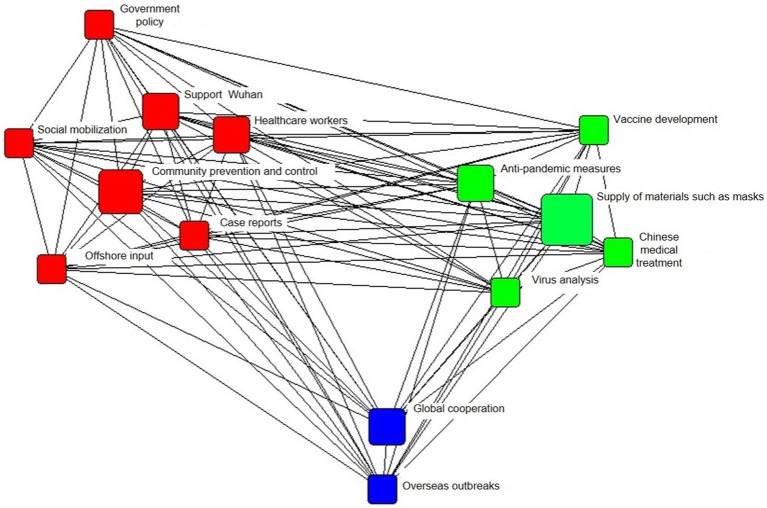
The network of the implicit public agenda.

Figure 5 shows the network visualization of the implicit public agenda in the COVID-19 pandemic. According to the degree centrality as shown in Table 8, the central theme was “supply of masks and other materials” (4,249), followed by “support Wuhan” (4,030), and “healthcare workers” (3,951).

**Table 8:** Implicit public agenda topic ranking.

Ranking	Topics	Degree centrality
1	Supply of materials such as masks	4,249
2	Support Wuhan	4,030
3	Healthcare workers	3,951
4	Vaccine development	3,713
5	Case reports	3,498
6	Anti-pandemic measures	3,357
7	Community prevention and control	3,203
8	Offshore input	3,029
9	Government policy	2,997
10	Virus analysis	2,975
11	Overseas outbreaks	2,332
12	Social mobilization	2,211
13	Global cooperation	1857
14	Chinese medicine treatment	1,278

Compared with the media agenda network, the public agenda paid more attention to “support Wuhan” and “case reports,” which were at the center of the network, and less attention to “virus analysis” and “overseas outbreaks.”

## Discussion and conclusion

Using longitudinal analysis, the current study found consistently significant correlations among different inter-media agenda networks during the COVID-19 pandemic. Early on in agenda-setting research in general, the Chapel Hill study already found a high degree of homogeneity among media agendas (Vu et al., 2014), and the current study similarly demonstrated that different media also have a high degree of homogeneity at the network agenda setting level, and that in the new media era, content across different media also has a high degree of mutual influence.

Furthermore, the current study proves that there was a significant correlation between media agendas and both explicit and implicit public agendas during the COVID-19 pandemic, which indicates that the media agenda network and the public agenda are still highly correlated in the new media era. Theoretically, this study complements the empirical research on agenda-setting in the Chinese context, and practically examines the effect of media influence on the cognitive, attitudinal, and behavioral levels of the media audience. At the same time, the NAS model also inspires the media to establish connections between various elements in their reporting, and the networked information structure helps to better deliver messages to the public, influence public opinions, and guide public sentiment in the new media era.

The media agenda network is more relevant to the implicit public agenda than it is to the explicit agenda, which is also consistent with previous findings, suggesting that the media agenda is more relevant to the automatic, unconscious, implicit public agenda than it is to the directly controlled, conscious, explicit public agenda. The difference between the two is determined by the fact that the media information is weakened after going through the “barrier” of the brain’s conscious information processing system. Because of our enhanced autonomy, humans are more likely to believe the information that our brains have chosen to hang onto after digesting it rather than to broadly accept all information immediately put out by media. The more directly the information is sent, the more likely the public is to be suspicious. This therefore offers suggestions as to how news media can communicate better, as truly effective communication is about outputting “logic,” which refers to prior interventions in the formation of public attitudes and motivations, not just “opinions” directly exported by the media. In the long run, this can also influence audience’s thinking, and help the audience form more rational logic through external positive guidance.

ChinaNews.com and Southern Metropolis Daily far exceeded cn-healthcare.com in terms of their posting volume, influence, and topic coverage, so the correlation between their agendas and the public agendas should have been higher than that of We Media like cn-healthcare.com. However, this study found that the latter had a significant correlation with both explicit and implicit public agendas, and the degree of correlation between the two was higher than that of the other two media and public agenda networks. This is a new finding in contrast to those of previous studies. While past studies have found that mainstream media plays an important role in guiding the public agenda, the power of We Media cannot be underestimated, particularly in the context of a pandemic. This is also closely related to the specific area of the COVID-19 pandemic, which is a public health issue, and cn-healthcare.com, as a vertical specialized We Media, has been able to meet public needs better in terms of dissemination of information regarding pandemic prevention, science, and treatments, which highlights the case of the audience reverse agenda-setting through a certain degree of selective exposure to media information. From a practical perspective, our findings can serve as useful input for public health communication during pandemic or similar situations. Apriliyanti et al. (2021) found that the media might help set the tone for policy agenda. Media can promote the interaction between science and policy. Policy makers and relevant news media should define a clear strategy as how to use social media during the pandemic. They should have a plan to know which topics are more critical to the public and work to guide them onto the public agenda (Tahamtan et al., 2022). For example, prolonged isolation can have a negative impact on physical and mental health. Based on the high level of public concern about which media to follow that can grow in response to strained physical or mental health stresses, news coverage should focus on accuracy of sources and issues such as vaccine development to ease public anxiety (Gong et al., 2022).

The media market is becoming highly segmented, and media consumption is becoming more personalized. Therefore, more attention must be paid to the role of the public agenda. Compared to the media agenda network, our findings showed that the public paid more attention to “support for Wuhan” and “case reports,” which were located at the center of the network. In the face of disasters, emotions are mobilized first due to many factors. Through media coverage of a series of deeds done in support of Wuhan, the public was inspired through strong emotions, and “when one side is in trouble, support from all sides” as a national spirit triggered a collective emotional resonance. This emotional narrative also offers suggestions for media communication strategies. These nationalistic emotions led to a strong and powerful portrayal of the role of health care workers fighting against the COVID-19 virus, which promoted solidarity, prosociality, and benevolence among the masses (Wang, 2022).

As individuals, however, the public is also prone to being extremely concerned about events that are closely related to their own lives. The “notification of cases” topic informed the public where outbreak was occurring and the number of confirmed cases in a clear and concise manner, saving the public time in accessing information. The public was then able to perceive and judge the seriousness of the outbreak, which in turn affected whether they would take effective measures to avoid a worse situation. Therefore, “case reports” was also a highly regarded topic by the public as an early warning device. This also demonstrates that in times of crisis, people’s need for orientation is very high. Previous studies have shown that people will borrow terms from media discourse to construct an articulate, relevant discussion because these ideas and terms have been previously validated by the media (Buturoiu and Gavrilescu, 2021). Therefore, before the public has the opportunity to manufacture doubts or speculation about virus outbreaks, the role of the media should be to set the tone for the nature of the outbreak. It is better for media to filter information and guide the public to understand key messages so that they can avoid becoming overwhelmed by the threat of pandemic information.

In this study, LDA Topic Modeling has been used for topic clustering of text content, which overcomes the flaws of traditional content analysis and uses machine learning for big data analysis and topic clustering with better results in matrix construction.

There are, nonetheless, some shortcomings to this study. In terms of the public agenda data, the sample size for the questionnaire was small; a larger sample should be used in the future. Second, in addition to examining media coverage topics, more dimensions could be added as measures, such as sentimental dimensions or positive and negative sentiment towards key individuals or events, to examine the degree of correlation between media agenda networks and public networks. Finally, this study only proved the existence of significant correlations among different media, and did not measure the agenda-setting causality among media and further than this, which should be tested in the future by using the Granger causality test to detect causality between the different media.

## Data availability statement

The original contributions presented in the study are included in the article/supplementary material; further inquiries can be directed to the corresponding author.

## Ethics statement

For social sciences, most institutions in China do not have Institutional Review Board. As a protection of all participants, the studies involving human participants were reviewed and approved by Beijing Jiaotong University, China. All participants voluntarily made their decision and consent to participate in this study.

## Author contributions

KL, XG, and XL: originated and designed research. XL and XG: contributed to the statistical analysis, interpretation of the results, and revision of the manuscript. All authors were involved in editing, reviewing, and providing feedback for this manuscript.

## Funding

This study was supported by the Fundamental Research Funds for the Central Universities (No. 2018JBW017).

## Conflict of interest

The authors declare that the research was conducted in the absence of any commercial or financial relationships that could be construed as a potential conflict of interest.

## Publisher’s note

All claims expressed in this article are solely those of the authors and do not necessarily represent those of their affiliated organizations, or those of the publisher, the editors and the reviewers. Any product that may be evaluated in this article, or claim that may be made by its manufacturer, is not guaranteed or endorsed by the publisher.

## Footnotes

^1^ ChinaNews.com

^2^ cn-healthcare.com

## References

[ref1] Apriliyanti I. D. Utomo W. P. Purwanto E. A. (2021). Examining the policy narratives and the role of the media in policy responses to the covid-19 crisis in Indonesia. J. Asian Public Policy 14, 1–17. doi: 10.1080/17516234.2021.1954770

[ref2] Buturoiu D. R. Gavrilescu M. (2021). Key words associated with the covid-19 pandemic. Comparing the media and the public agenda. J. Media Res. 14, 5–25. doi: 10.24193/jmr.40.1

[ref4] Chen A. Zhang X. (2021). Changing social representations and agenda interactions of gene editing after crises: a network agenda-setting study on Chinese social media. Soc. Sci. Comput. Rev. 40, 1133–1152. doi: 10.1177/0894439321998066

[ref5] China Internet Network Information Center (2021). The 47th China statistical report on Internet development. Available at: http://www.cnnic.net.cn/hlwfzyj/hlwxzbg/ (Accessed August 10, 2022).

[ref6] Golan G. Wanta W. (2001). Second-level agenda setting in the New Hampshire primary: a comparison of coverage in three newspapers and public perceptions of candidates. J. Mass Commun. Q. 78, 247–259. doi: 10.1177/107769900107800203

[ref7] Gong J. K. Firdaus A. (2022). Is the pandemic a boon or a bane? News media coverage of COVID-19 in China daily. Journal. Pract. 16, 1–21. doi: 10.1080/17512786.2022.2043766

[ref8] Gong J. Firdaus A. Said F. Ali Aksar I. Danaee M. Xu J. (2022). Pathways linking media use to wellbeing during the COVID-19 pandemic: a mediated moderation study. Soc. Media Soc. 8:205630512210873. doi: 10.1177/20563051221087390

[ref10] Guo L. (2012). The application of social network analysis in agenda setting research: a methodological exploration. J. Broadcast. Electron. Media 56, 616–631. doi: 10.1080/08838151.2012.732148

[ref11] Guo L. Mays K. Wang J. N. (2019). Whose story wins on twitter? Visualizing the South China Sea dispute. Journal. Stud. 20, 563–584. doi: 10.1080/1461670X.2017.1399813

[ref12] Guo L. McCombs M. (2011). “*Toward the third level of agenda setting theory: a network agenda setting model*” in *Paper presented at the AEJMC Annual Conference*.

[ref13] Guo L. Vargo C. J. (2015). The power of message networks: a big-data analysis of the network agenda setting model and issue ownership. Mass Commun. Soc. 18, 557–576. doi: 10.1080/15205436.2015.1045300

[ref14] Guo L. Vargo C. J. Pan Z. Ding W. Ishwar P. (2016). Big social data analytics in journalism and mass communication: comparing dictionary-based text analysis and unsupervised topic modeling. J. Mass Commun. Q. 93, 332–359. doi: 10.1177/1077699016639231

[ref16] Hanneman R. A. Riddle M. (2005). Introduction to Social Network Methods. California: University of California

[ref17] Jiang Q. L. Cheng Y. (2018). The third-level agenda setting: media and public agenda networks during THAAD event. Chin. J. Commun. 40, 85–100. doi: 10.13495/j.cnki.cjjc.2018.09.005

[ref18] Jiang Q. L. Cheng Y. Cho S. K. (2021). Media coverage and public perceptions of the THAAD event in China, the United States, and South Korea: a cross-national network agenda-setting study. Chin. J. Commun. 14, 386–408. doi: 10.1080/17544750.2021.1902360

[ref20] Kweon S.-H. Go T. Kang B. Cha M.-K. Kim S.-J. Kweon H.-J. (2019). Study on agenda-setting structure between SNS and news: focusing on application of network agenda-setting. Int. J. Contents. 15, 10–24. doi: 10.5392/IJOC.2019.15.1.010

[ref21] McCombs M. (2005). A look at agenda-setting: past, present and future. Journal. Stud. 6, 543–557. doi: 10.1080/14616700500250438

[ref22] McCombs M. Llamas J. P. Lopez-Escobar E. Rey F. (1997). Candidate images in Spanish elections: second-level agenda-setting effects. J. Mass Commun. Q. 74, 703–717. doi: 10.1177/107769909707400404

[ref25] Schultz F. Kleinnijenhuis J. Oegema D. Utz S. Van Atteveldt W. (2012). Strategic framing in the BP crisis: a semantic network analysis of associative frames. Public Relat. Rev. 38, 97–107. doi: 10.1016/j.pubrev.2011.08.003

[ref28] Su Y. Hu J. (2020). A territorial dispute or an agenda war? A cross-national investigation of the network agenda-setting (NAS) model. J. Inf. Technol. Polit. 17, 357–375. doi: 10.1080/19331681.2020.1756553

[ref29] Sung M. Hwang J. S. (2014). Who drives a crisis? The diffusion of an issue through social networks. Comput. Hum. Behav. 36, 246–257. doi: 10.1016/j.chb.2014.03.063

[ref30] Tahamtan I. Potnis D. Mohammadi E. Singh V. Miller L. E. (2022). The mutual influence of the World Health Organization (WHO) and twitter users during COVID-19: network agenda-setting analysis. Med. Internet Res. 24:e34321. doi: 10.2196/34321, PMID: 35275836 PMC9045487

[ref31] Vargo C. J. Guo L. (2017). Networks, big data, and intermedia agenda setting: an analysis of traditional, partisan, and emerging online U.S News. J. Mass Commun Q. 94, 1031–1055. doi: 10.1177/1077699016679976

[ref33] Vargo C. J. Guo L. Mccombs M. Shaw D. (2014). Network issue agendas on twitter during the 2012 U.S. presidential election. J. Commun. 64, 296–316. doi: 10.1111/jcom.12089

[ref34] Vu H. T. Guo L. McCombs M. E. (2014). Exploring “the world outside and the pictures in our heads”: a network agenda-setting study. J. Mass Commun. Q. 91, 669–686. doi: 10.1177/1077699014550090

[ref35] Wang Q. (2016). A comparative case study: network agenda setting in crisis and non-crisis news. Glob. Media Commun. 1, 208–233. doi: 10.1177/2059436416668870

[ref36] Wang Q. (2022). Using social media for agenda setting in Chinese government’s communications during the 2020 COVID-19 pandemic. J. Commun. Inq. 46, 373–394. doi: 10.1177/01968599221105099 PMC9157277 38603167

[ref37] Wang X. H. Chen L. Shi J. Y. Tang H. J. (2021). Who sets the agenda? The dynamic agenda setting of the wildlife issue on social media. Environ. Commun. 15, 1–18. doi: 10.1080/17524032.2021.1901760

[ref38] Wang H. X. Shi J. (2022). Intermedia agenda setting amid the pandemic: a computational analysis of China's online news. Comput. Intell. Neurosci. 17, 357–375. doi: 10.1080/19331681.2020.1756553 PMC9012972 35437439

[ref39] Weaver D. H. Shaw D. Wojdynski B. McKeever R. (2010). “Vertical and or versus? Horizontal communities: Need for orientation, media use and agenda melding,” in *Paper presented at the Annual Conference of the World Association of Public Opinion Research*.

[ref40] Weiss-Blatt N. (2016). “Tech bloggers vs. tech journalists in innovation journalism,” in *Paper presented at 2016 3rd European Conference on Social Media*, 415–423.

